# Diseases of the Kidney

**Published:** 1903-03-14

**Authors:** 


### Diseases of the Kidney.

(*Concluded from page* 393.)

**Uræmia.**—Morphia has been advocated by some authors in the treatment of delirium and convulsions and regarded as a poison by others. Edgeworth and Carter[4] report a case of chronic uræmia in granular kidney where the drug was used for two years with most favourable results in numerous attacks.

**Venesection in acute nephritis** is discussed by R. W. Marsden,[5] who gives a short but interesting account of the change of fashion as to bleeding at various epochs. He points out that bleeding is likely to be efficacious either for relieving an overburdened right heart or for removing a poison from the blood when that poison interferes with its oxygen carrying powers and is not combined with the tissues. If it is, an alkaloid combination takes place and bleeding cannot abstract it. This he thinks is the case in uræmia and the simultaneous injection of saline fluid would, he adds, also fail to remove the poison. No doubt the injection of saline fluid acts as a diuretic, but this injection may be given per rectum without venesection except when there is urgent collapse. On the whole venesection is useful only from its action on the circulation by relieving the heart and enabling it to maintain the needful pressure in the kidneys. It will not remove the poisons causing uræmia. The use of saline injections combined with a milk diet, sudorifics, and cathartics is recommended by a writer in Merck's Archives. A pint of normal saline solution is to be given per rectum three times a day from a fountain through a long rectal tube or canula. This should enter the sigmoid flexure. The patient should be on his right side with the buttocks raised. The solution should be at the body temperature. Others recommend a temperature of 120° and a double canula, that a continuous flow in and out of hot water may be obtained and act as a local stimulant to the kidneys. In either case it is important that the fluid should be made to pass up as high as possible. The lower bowel should be first emptied by an ordinary enema, and if the pint injections of saline fluid are used care must be taken that they are retained and absorbed.

**Tuberculosis.**—P. Newmark[6] thinks the most frequent route of infection of the bladder is from the urine through the kidneys; the next is by direct extension from the ureter to the bladder. Rarer sources are from the prostate, seminal vesicles and even from the blood stream. The difficulties of diagnosis are great. There are very frequent calls to micturition, with pain in the middle of the urethra and not at the meatus, the pain coming on before micturition and continuing afterwards. There is hæmaturia, merely tinting the urine, unless ulceration occurs, and tubercle bacilli in the urine. To discover the latter the urine must be centrifugalised soon after it is passed, and even then they may be so few that none may be found. The smegma bacilli are found in spite of all precautions, such as sterilised catheters, but they are decolorised by acid alcohol and never lie in groups of parallel rows as tubercle bacilli often do. For treatment he advises the general measures used for phthisis with guiacol or its carbonate increased up to a drachm of either preparation daily and then reduced. Icthyol may be tried up to three drachms, and if pus is present, salol, resorcin, methylene blue, or boric acid can be given. Local treatment should be avoided, though some good results seem to have been obtained with instillations of corrosive sublimate in small quantities. Tuberculosis of the kidney may be acute and miliary, which is nearly always bilateral; or it may be caseous or of the pyelonephritic type. Here, too, pain, hæmaturia, and enlargement of the kidney are insufficient for diagnosis unless the bacilli are also found. (However, H. Walsham has shown that bacilli may be excreted through the kidney without the formation of tubercles in it, and Foulerton and Hillier have proved the excretion of virulent bacilli in nine cases where post-mortem no tuberculosis of the urinary tract could be found.) By cystoscopy, too, a diagnosis may sometimes be made, though it is undesirable to use this or any other instrumental procedure in the bladder if it can be avoided. In a doubtful case the injection of tuberculin has led to a correct decision by the general reaction and the tenderness and pain in the affected kidney and ureter. If we find definite disease of the kidney surgical aid is usually needed; but here, too, it is important to decide whether one kidney is healthy. Henry Morris holds that occasionally well-marked cases have been cured by climate and diet, but the prognosis is usually most unfavourable unless the disease is strictly localised and the affected area completely removed. The caseous form is often of long duration, becoming quiescent at times and going on sometimes for several years. The presence of suppuration or severe hæmorrhages hastens the end. The mortality after nephrectomy has been greatly reduced of late, so that Tilden Brown claims to have brought it down to 7 per cent. ("Surgical Diseases of Kidney.")

**Myelopathic Albumosuria.**—T. R. Bradford[7] remarks that under the term osteomalacia there were formerly included not only cases of recoverable softening in young women, but also what is now called multiple myeloma. Among the latter cases a special group may be separated, which presents Bence Jones' albumen in the urine and some affection of the marrow. Only about 20 instances of this type have been recorded. The patients are generally males in the second half of life. The ribs sternum, vertebræ, pelvic bones and skull are invaded by a new growth which absorbs the bony tissue and is of the appearance of red marrow. It is composed of round cells and is closely allied to sarcoma, but does not form metastases elsewhere. The albumose coagulates at a low temperature or on the addition of nitric acid, but re-dissolves on boiling. Hydrochloric acid also coagulates it. Severe pains in the back or sides with anæmia and weakness appear at an early stage of the disease, while the bones may become soft and pliable or show the presence of tumours. The progress of the disease is steadily downhill. The proteid is not hetero-albumose but a definite body which stands between true albumen and proto-albumose, but the mode of its production is uncertain. Possibly some abnormal metabolism is caused by a ferment derived from the new growths. The origin of the ordinary albumen found in the urine has had some light thrown on it by the new tests for precipitins. When a definite proteid is injected into the vessels of animals, a body is formed in the blood which will precipitate a solution containing that proteid and no other. Mertens found that injections of blood caused the appearance of a body which precipitates urinary albumen, and he argued therefore that the albumen is derived from blood. L. Aschoff[8] further shows that the albumen is not derived from the kidney epithelium, though his experiments are not entirely conclusive.

**Alkaptonuria.**—A. E. Garrod[9] remarks that the constant factor in this condition is the excretion of homogentisic acid which causes the darkening of the urine with alkalies and on exposure to the air, the reduction of metallic salts and the staining of fabrics. The other acid occasionally present, uroleucic, is not found without the first-named one, and seems to disappear in later life. The condition is probably not a disease, but an alternative form of metabolism, usually congenital and lifelong, and perhaps slightly inferior to the normal method. It is most frequent in males, and especially in the children of first cousins, but only one instance of transmission from parent to offspring is on record. If it is an occasional "sport" the marriage of two persons, in whom there is an hereditary tendency of the kind, affords the best chance of its development. Albinism, a similar chemical abnormality, tends likewise to appear in males or in brothers and sisters of families in which it has been absent for some generations. There is some evidence, too, of its frequency in the children of first cousins. A third condition, probably of the same hereditary kind, is seen in cystinuria, though this may produce harm from the risk of formation of calculi. Differences in metabolism and chemical processes are seen between many species of animals, such as their different reaction to poisons, and the variation of their excretions, and minor differences within a species, such as the colour of hair and eyes, are of course very frequent, so that an abnormal mode of metabolism, such as this view of alkaptonuria suggests, is not without parallels.

4 Bristol Med. Chir. Jour., Sept. 5 Med. Chron., Oct 6 Med. Rec., Sept. 27. 7 Lancet, Oct. 4. 8 Ibid, Sept. 6. 9 Ibid. Dec. 13.

